# Targeting choroidal vasculopathy via up-regulation of tRNA-derived fragment tRF-22 expression for controlling progression of myopia

**DOI:** 10.1186/s12967-023-04274-5

**Published:** 2023-06-24

**Authors:** Chang Liu, Meiyan Li, Yaming Shen, Xiaoyan Han, Ruoyan Wei, Yunzhe Wang, Shanshan Xu, Xingtao Zhou

**Affiliations:** 1 Eye Institute and Department of Ophthalmology, Eye & ENT Hospital, Fudan University, Shanghai, 200031 China; 2 NHC Key Laboratory of Myopia (Fudan University), Key Laboratory of Myopia, Chinese Academy of Medical Sciences, Shanghai, 200031 China; 3 Shanghai Research Center of Ophthalmology and Optometry, Shanghai, 200031 China; 4 Shanghai Key Laboratory of Visual Impairment and Restoration, Shanghai, 200031 China; 5 The Affiliated Eye Hospital, Nanjing Medical University, Nanjing, 210029 China; 6 The Fourth School of Clinical Medicine, Nanjing Medical University, Nanjing, 210029 China

**Keywords:** Myopia, Choroidal vasculopathy, Transfer RNA-derived fragment, METTL3, m^6^A modification

## Abstract

**Background:**

Myopia has emerged as a major public health concern globally, which is tightly associated with scleral extracellular matrix (ECM) remodeling and choroidal vasculopathy. Choroidal vasculopathy has gradually been recognized as a critical trigger of myopic pathology. However, the precise mechanism controlling choroidal vasculopathy remains unclear. Transfer RNA-derived fragments (tRFs) are known as a novel class of small non-coding RNAs that plays important roles in several biological and pathological processes. In this study, we investigated the role of tRF-22-8BWS72092 (tRF-22) in choroidal vasculopathy and myopia progression.

**Methods:**

The tRF-22 expression pattern under myopia-related stresses was detected by qRT-PCR. MTT assays, EdU incorporation assays, Transwell migration assays, and Matrigel assays were conducted to detect the role of tRF-22 in choroidal endothelial cell function in vitro. Isolectin B4 staining and choroidal sprouting assay ex vivo were conducted to detect the role of tRF-22 in choroidal vascular dysfunction in vivo. Immunofluorescent staining, western blot assays and ocular biometric parameters measurement were performed to examine whether altering tRF-22 expression in choroid affects scleral hypoxia and ECM remodeling and myopia progression in vivo. Bioinformatics analysis and luciferase activity assays were conducted to identify the downstream targets of tRF-22. RNA-sequencing combined with m6A-qPCR assays were used to identify the m6A modified targets of METTL3. Gain-of-function and Loss-of-function analysis were performed to reveal the mechanism of tRF-22/METTL3-mediated choroidal vascular dysfunction.

**Results:**

The results revealed that tRF-22 expression was significantly down-regulated in myopic choroid. tRF-22 overexpression alleviated choroidal vasculopathy and retarded the progression of myopia in vivo. tRF-22 regulated choroidal endothelial cell viability, proliferation, migration, and tube formation ability in vitro. Mechanistically, tRF-22 interacted with METTL3 and blocked m^6^A methylation of Axin1 and Arid1b mRNA transcripts, which led to increased expression of Axin1 and Arid1b.

**Conclusions:**

Our study reveals that the intervention of choroidal vasculopathy via tRF-22-METTL3- Axin1/Arid1b axis is a promising strategy for the treatment of patients with myopic pathology.

**Graphical Abstract:**

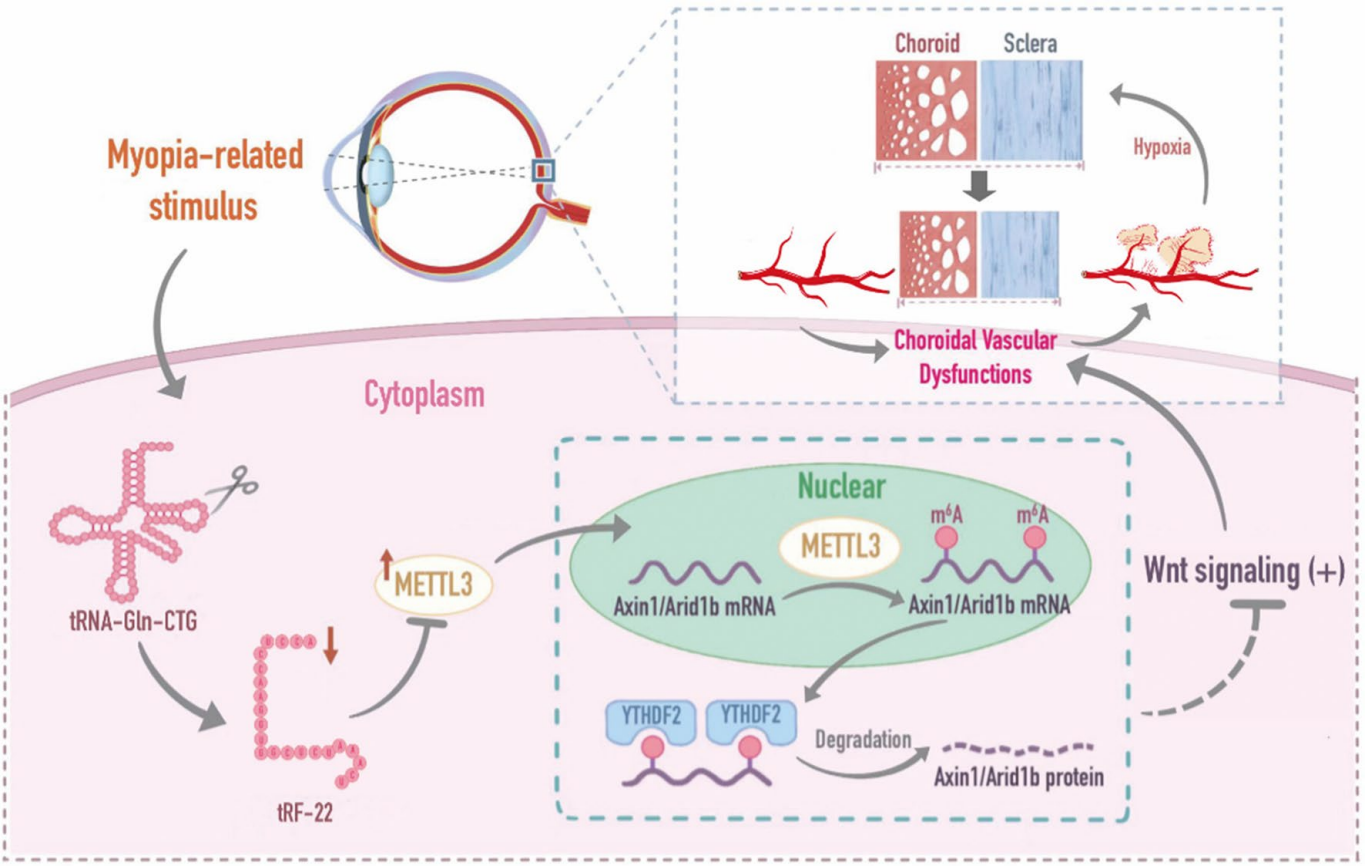

**Supplementary Information:**

The online version contains supplementary material available at 10.1186/s12967-023-04274-5.

## Background

Myopia has become a major public health concern. The prevalence of myopia is estimated to reach 4.949 billion by 2050 [1]. Myopia seriously impacts children and adolescents’ overall quality of life. Moreover, high myopia has been recognized as a risk factor for ocular diseases, such as choroidal neovascularization (CNV), retinal detachment, glaucoma, and macular atrophy, which may cause irreversible visual loss [2, 3]. Although considerable financial resources have been invested in improving refractive correction and slowing the progression of myopia, it’s still hard to halt the progression and minimize the complications of high myopia due to the ambiguity of pathogenesis [3–6]. Thus, exploring the potential mechanism underlying high myopia is still required for slowing the progression of myopia.

The thinning of sclera occurs is a typical pathological change during myopia progression, which results in scleral strength decline and ocular axial elongation. Substantial evidence has indicated that microenvironmental hypoxia act as a key driver for scleral remodeling [7–10]. The choroid is a highly vascularized intermediary between the retina and sclera, and provides oxygen to support the microenvironment homeostasis in the sclera [11, 12]. Choroidal vasculopathy can lead to scleral hypoxia during the progression of myopia. Therefore, the choroid may hold promise as a therapeutic target for the prevention or retardation of myopic pathology. Despite numerous studies, the responsible molecular mechanisms of choroidal vasculopathy remain unknown in myopia.

During the progression of myopia, mechanical stress, hypoxia, inflammatory response, and oxidative stress lead to choroidal vascular dysfunction [13, 14]. Transfer RNA-derived fragments (tRFs), a novel and ubiquitous class of non-coding RNAs, are derived from tRNA following endonuclease cleavage, which occurs under various stress conditions [15, 16]. In the context of stresses by different enzymes or proteins such as Dicer, angiogenin, and RNase Z, tRFs have been discovered in numerous cell types and organisms and play important roles in a variety of biological processes [17–20]. For instance, through interacting with particular proteins or mRNAs, tRFs control cell survival, death, metastasis, and differentiation [21, 22]. In addition, tRFs are closely associated with the initiation, development, and prognosis of various vascular diseases via regulating Notch, Wnt/β-catenin, and PI3K/Akt signaling [23–25]. Thus, we speculate that myopia-related pathologic stimuli may alter the expression of tRFs, which in turn affect choroidal vascular homeostasis.

In this study, we determined the role of tRF-22-8BWS72092 designated as tRF-22, in choroid vasculopathy and the progression of myopia and clarified the underlying mechanism of tRF-22-mediated choroid vasculopathy This study would provide novel insights into the regulation of choroidal vasculopathy and provided a new strategy for the prevention and management of myopia.

## Methods

### Animal experiment

All animal experimental procedures were performed with the approval of the Animal Care and Use Committee of Eye & ENT Hospital (Shanghai, China) and adhere to the Association for Research in Vision and Ophthalmology (ARVO) Statement for the Use of Animals in Ophthalmic and Vision Research. Pigmented guinea pigs or C57BL/6 J mice were purchased from Shanghai Laboratory Animal Center (Shanghai, China). They were reared with a 12-h light–dark cycle and maintained with 40–60% humidity at room temperature (25 ± 1 °C).

### Induction of form-deprived myopia (FDM)

Form-deprived myopia was induced as shown below [26]. Briefly, 3-week-old male pigmented guinea pigs or 3-week-old male C57BL/6 J mice were used for the induction of form-deprived (FD) myopia. Myopia was induced by the placement of a translucent eye shield onto one eye for 2-, 4-, and 8-week. The contralateral untreated eye served as the control. After 2-, 4-, and 8-week FD treatment, the eye shield was removed. The biometric measurements were performed in a dark environment. A streak retinoscopy (66 Vision Tech, Jiangsu Province, China) was used to measure refractive errors and trial lenses on awake guinea pigs. A-scan ultrasonography device (Kangning, China) was used to measure ocular biometric parameters, including VCD and AL.

### Choroidal neovascularization model

Male C57BL/6 J mice (6 to 8 weeks of age) were used for building the choroidal neovascularization model. Briefly, they were anesthetized by ketamine (80 mg/kg) plus xylazine (10 mg/kg) mixture. The pupils were dilated with 1% tropicamide and four spots were created at a distance of approximately three times the optic disc diameter from optic nerve head. The four spots were evenly distributed and avoided vessels. The laser settings were shown below: spot size, 100 μm; duration, 50 ms; and power, 120 mW. The bubbles formation indicated that Bruch’s membrane was disrupted. After laser photocoagulation, the mice were placed on a heat lamp for recovery until they fully awaken.

### Immunofluorescent staining

After the required treatment, the eyeballs were dissected and placed in Fekete’s solution at 4 °C for 2 h. Then, the cornea and lens were removed. The remaining tissues were fixed in 4% paraformaldehyde (PFA) at 4 °C overnight, implanted in OCT compound after being dehydrated with a 30% sucrose solution overnight, and stored at − 80 °C until sectioning. The frozen samples were sectioned on a cryostat at − 25 °C to a thickness of 5 μm and placed on the adhesion microscope slides (Citotestt, China). The frozen sections were washed with PBS 3 times, permeabilized, and blocked with 5% BSA for 30 min at room temperature. The slides were incubated with the following primary antibodies, including collagen type I (1:200, ab21286, Abcam), α-SMA (1:500, 48938S, Cell Signaling Technology), and vinculin (1:500, ab129002, Abcam) for at 4 °C overnight. After washing with PBST, the secondary antibodies were added, including goat anti-rabbit IgG (H + L) (1:500, A11012, Invitrogen) and goat anti-mouse IgG (H + L) (1:500; A11005, Invitrogen) at room temperature for 2 h. DAPI (1:1500, C1002, Beyotime) was finally added to label cell nuclei for 10 min. The staining results were observed under an Olympus IX73 inverted microscope (Olympus, Japan).

### Hematoxylin and eosin (HE) staining

To perform a histochemical analysis of the neovascularization in choroid after laser injury, HE staining was conducted as follows. The eyeballs were enucleated, removed corneas and lenses, then fixed in 4% paraformaldehyde (PFA) overnight at 4 °C. The fixed samples were dehydrated in graded ethanol, embedded in paraffin and cut vertically through the optic nerve head to a thickness of 5 μm. Following dewaxed and rehydrated, the sections were stained with hematoxylin for 1.5 min, washed with double-distilled water 3 times, and stained with Eosin for 50 s. After staining, samples were dehydrated with 100% ethanol, cleared in xylene, and fixed with neutral resin. Images were taken and the thickness of the lesion from the bottom of the pigmented choroidal layer to the top of the neovascular membrane was measured with Image J.

### Fluorescein angiography (FA)

The choroidal neovascularization leakage was assessed via FA (10 d after laser induction). After pupil dilatation, fundus photographs were taken to visualize the site and effective presence of laser burn, then, 0.1 ml of 10% fluorescein in saline was injected intraperitoneally and fluorescent fundus images were acquired at 1–3 and 5–7 min respectively. Two blinded observers evaluated the increase in size/intensity of dye between the early and late phases, and the fluorescein leakage was graded qualitatively according to the following criteria: grade 0, no hyperfluorescence; grade 1, slight hyperfluorescence with no increase in intensity nor size; grade 2, hyperfluorescence increasing in intensity but not in size; grade 3, hyperfluorescence increasing both in intensity and size; grade 4, hyperfluorescence size increase more than 2-diameter of the initial laser burn.

### Choroid sprouting assay

After 4-week-form deprivation, the eyes of mice were removed and preserved in the ice-cold DMEM until being dissected. After removing the cornea and lens, choroid and retinal pigment epithelium (RPE) explants were separated and sliced approximately into 1 mm^2^ piece. Subsequently, the explants were then added to 24-well plates filled with 300 μl Matrigel (BD Biosciences, USA) in 24-well plates. The culture medium was changed every 2 days. Each sample was photographed on day 4, 5, and 6, respectively. The sprouting area was calculated by Image J software.

### Quantitative real-time PCR (qRT-PCR)

Total RNAs were isolated by the TRIzol reagent (Invitrogen, USA) and determined by the NanoDrop 1000 instrument (Nanodrop Technologies, USA). 1 μg of total RNAs was used for reverse transcription by HiScript III RT SuperMix (Vazyme, China) and mRNAs were quantified by qRT-PCR assays using ChamQ Universal SYBR qPCR Master Mix Kit (Vazyme, China). tRF-RNAs were extended the 3’ end through Poly(A) addition using Poly(A) Polymerase in reverse transcription reaction (microRNA Reverse Transcription Kit, EZBioscience, USA) and then quantified by probe based real-time qRT-PCR using EZ-Probe qPCR Master Mix for microRNA (ROX2 plus) Kit (EZBioscience, USA). The sequence-specific forward primers of tRF-RNAs were as follows: tRF-22: 5’-GGGTCAAATCTCGGTGGAAC-3’, U6: 5’-CCTGCTTCGGCAGCACA-3’. The probes and reverse primers were included in the EZ-Probe qPCR Master Mix for microRNA (ROX2 plus) Kit. The primers of mRNAs were exhibited in Additional file 1: Table S1. Each reaction was performed at least in triplicate and the 2^−∆∆Ct^ method was used for quantitative analysis.

### Northern blot

Northern blot was performed as previously described [27]. In brief, 20 μg of total RNAs were isolated with TRIzol Reagent (Invitrogen, USA), separated on the denaturing polyacrylamide gel, and transferred to a nylon membrane. After the pre-hybridization, the membrane was hybridized with oligonucleotide probes labeled with biotin, the probe sequence was as followed: 5’-TGGAGGTTCCACCGAGATTTGA-3’. The probes were detected with the Chemiluminescent Biotin-labeled Nucleic Acid Detection Kit (YaJi Biological, China).

### Quantification of m^6^A level

m^6^A levels were assayed by the m^6^A RNA Methylation Quantification Kit (Abcam, UK). In brief, the negative control RNA, positive control RNA, or the experimental RNA sample (200 ng) was added to each well and mixed with the high binding solution, respectively. m^6^A was captured and assayed using the diluted concentration of capture and detection antibody solution. After incubation with the enhancer and developer solution, m^6^A level was determined at the absorbance of 450 nm wavelength.

### m^6^A dot blot assay

mRNA fraction was isolated from total RNAs using the Dynabeads mRNA Purification Kit (Thermo Scientific, USA). Then, mRNA samples were spotted on the Amersham Hybond N^+^ membrane (GE Healthcare, USA), exposed to UV light for 5 min, blocked with 5% BSA for 1 h, incubated with m^6^A antibody (1:1000; 202,003, Synaptic Systems) overnight at 4 °C, and incubated with the goat anti-rabbit HRP-conjugated secondary antibody (1:2500, sc-2030, Santa Cruz Biotechnology) for 1 h. The staining results were visualized using a chemiluminescence detection system (Tanon, China).

### RNA immunoprecipitation assay

The Magna RIP RNA-Binding Protein Immunoprecipitation Kit (17–700, Millipore, USA) was used to conduct the RNA immunoprecipitation (RIP) according to the manufacturer's instruction. Briefly, the magnetic beads were used to collect the samples, and 5 g of either YTHDF2 (80,014, CST) or mouse immunoglobulin G (17–700, Millipore) were added. After using the magnetic separator to precipitate protein-RNA complexes, the supernatant was discarded and proteinase K was added to each immunoprecipitate. Finally, the total RNAs were extracted and qPCR assays were conducted to detect the interaction between YTHDF2 and Axin1 or Arid1b.

### Western blot

Total proteins were isolated from choroidal or scleral tissues by the RIPA Lysis buffer (Beyotime, China). The bicinchoninic acid (BCA) protein test kit (Beyotime, China) was used to detect protein concentration. About 50 µg of proteins were separated on 8% SDS-PAGE and then transferred to PVDF membranes (Millipore, USA). The membranes were blocked with 5% BSA and incubated with the primary antibodies: METTL3 (1:1000, 15,073–1-AP, Proteintech), collagen type I (1:1000, ab88147, Abcam), α-SMA (1:2000, 48938S, Cell Signaling Technology), vinculin (1:1000, ab129002, Abcam), Axin1 (1:1000, 2087S, Cell Signing Technology,), Arid1b (1:1000, ab226762, Abcam), β-actin (1:1000, sc-8432, Santa Cruz Biotechnology) and β-tubulin (1:2000, ab6046, Abcam) overnight at 4 °C. Then, the membranes were treated for 3 hat room temperature with the HRP-labeled goat anti-mouse IgG(H + L) (1:1000, A0216, Beyotime) and goat anti-rabbit IgG(H + L) (1:1000, A0208, Beyotime). The protein bands were visualized using the enhanced chemiluminescence detection system (Tanon, China).

### Cell culture

RF/6A cells were cultured in DMEM with 10% fetal bovine serum (Gibco, USA) and 1% penicillin–streptomycin solution (Gibco, USA). They were incubated in a sterile humidified incubator at 37 °C in 5% CO_2_. Before transfection, RF/6A cells were placed into the 24-well plates. When the confluence reached about 80%, lipofectamine 6000 (Beyotime, China) was used to transfect siRNAs into RF/6A cells in accordance with the manufacturer's protocols.

### Cell viability assay

To assess cell viability, MTT [3-(4, 5-dimethylthiazol-2-yl)-2, 5-diphenyltetrazolium bromide] assays were performed. Briefly, RF/6A cells were seeded in a 96-well plate. Following the necessary treatment, 10 μl of MTT solution (5 mg/ml, Sigma-Aldrich, USA) was added into each well and incubated for 4 h at 37 °C. The formazan crystals were dissolved in 100 μl of DMSO. A microplate spectrophotometer (Bio-Tek Instruments Inc., USA) was used to determine the absorbance at 490 nm.

### Cell proliferation assay

The 5-ethynyl-2'-deoxyuridine (EdU) assay kit (Ribobio, China) was utilized to detect the proliferation ability of RF/6A cells. Briefly, the cells were seeded in 24-well plates. After the necessary treatment, RF/6A cells were fixed with 4% paraformaldehyde for 30 min, permeabilized with 0.3% Triton X-100 for 20 min, and then incubated with EdU reagent (50 M) at 37 °C for 2 h. The cell nuclei were stained with DAPI (Sigma-Aldrich, USA) for 15 min in a dark chamber and the staining results were observed by an inverted fluorescence microscope (Carl Zeiss Meditec, Germany).

### Cell migration assay

Transwell plates (6.5 mm in diameter, 3422, Corning) with 8 μm pore filters were utilized to detect the migration ability of RF/6A cells. Briefly, approximately 2 × 10^4^ cells were seeded into the upper chamber and allowed to migrate into the bottom chamber. The migrated cells were fixed with 4% paraformaldehyde (Biosharp, China) and stained with 0.5% crystal violet solution. These non-migrated cells were removed by scraping and the migration rate was expressed as the fold change of the number of migrated cells through a Transwell plate.

### Tube formation assay

Tube formation assays were performed to evaluate the angiogenic ability of endothelial cells. Briefly, RF/6A cells (1 × 10^4^ cells/well) were seeded onto Matrigel-precoated 96-well plates (BD Biosciences, USA). Then, these cells were cultured at 37 °C for 8 h. Finally, the tube formation was observed using an inverted microscope (Nikon, Tokyo, Japan).

### Aqueous humor collection

A total of 15 myopic CNV (mCNV) patients and 15 controls (patients with cataracts) were recruited from the Eye and Ear Nose Throat Hospital, Fudan University. The inclusion criteria were first diagnosis with mCNV without laser photocoagulation, photodynamic therapy or anti-VEGF therapy. Patients with other ocular diseases or ocular infection were excluded. About 100–200 μL of aqueous humor from each eye was collected and stored at − 80 °C until analysis. Informed consent was obtained from all patients. Institutional Review Board of the authors’ institute approved this study and the study adhered to the tenets of the Declaration of Helsinki.

### Statistical analysis

Experimental data are presented as the mean ± SD. For two-group comparisons, the significance was determined by Student's *t*-test. For more than two group comparisons, the significance was determined by using one-way or two-way ANOVA followed by Bonferroni's post-hoc test. All statistical analysis was performed using GraphPad Prism 8. *P* < 0.05 was considered to be statistically significant.

## Results

### tRF-22 expression is down-regulated in the myopic choroid tissues

**Fig. 1:**
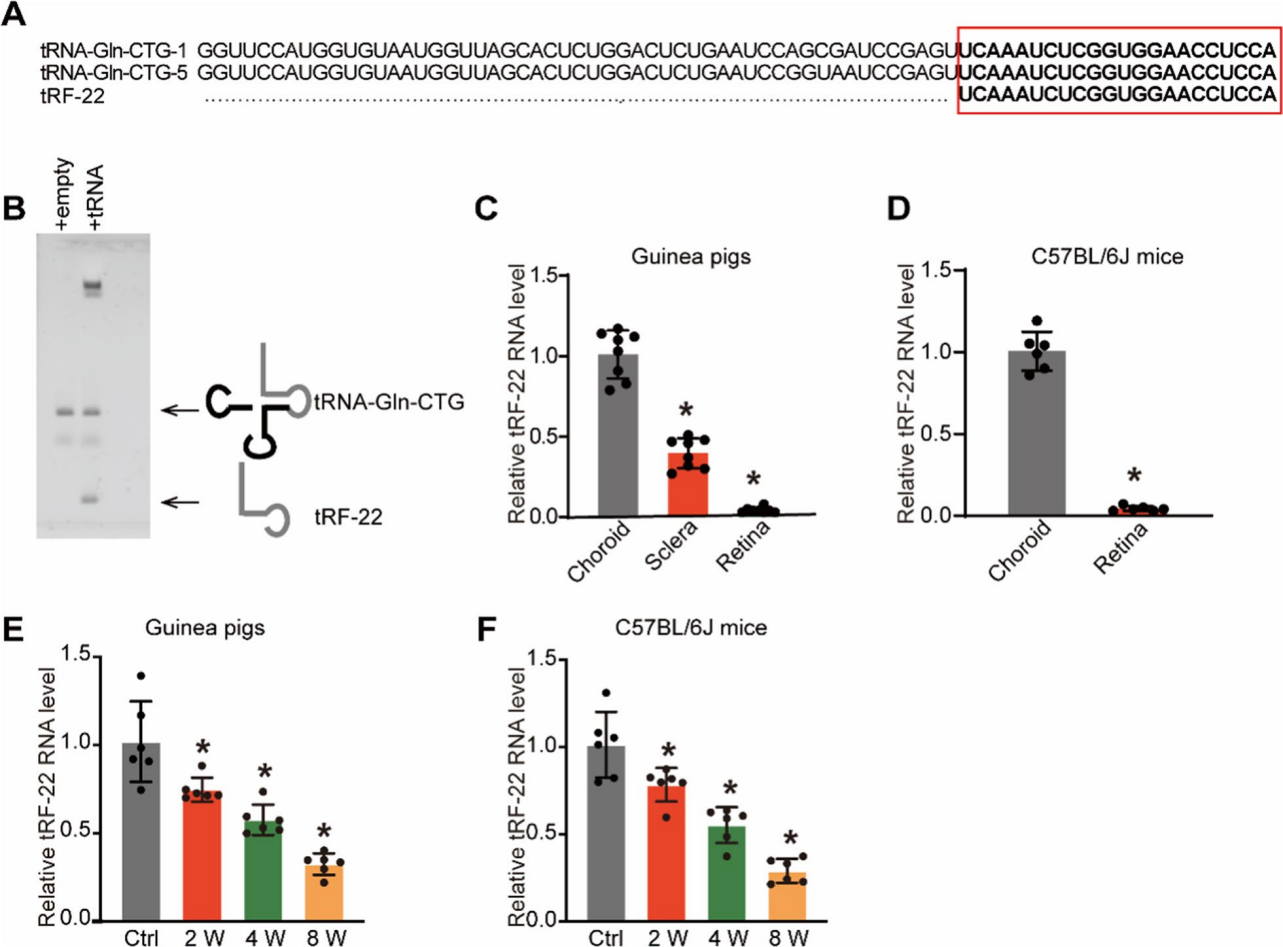
tRF-22 expression is down-regulated in the myopic choroid tissues. **A** The sequences of annotated human tRNAs (tRNA^Gln−CTG^) with perfect match to tRF-22. tRF-22 sequence is highlighted in bold. **B** Northern blot analysis of total RNAs from 293 T cells transiently transfected with an empty vector or a vector encoding for the tRNA^Gln−CTG^. The three primary bands correspond to 22-nt tRNA fragment tRF-22, 75-nt mature tRNA, and a high molecular weight tRNA primary transcript. **C** The three-week-old guinea pigs underwent monocular FD induction using the translucent eye shield. qRT-PCR assays were conducted to detect the expression of tRF-22 in the retina, sclera, and choroid tissue of guinea pigs (n = 6; *P < 0.05 vs. choroid group). **D** qRT-PCR assays were conducted to detect the expression of tRF-22 in the retina and sclera/choroid complex tissue of C57BL/6 J mice (n = 6; *P < 0.05 vs. choroid group). **E** qRT-PCR assays were conducted to detect the expression of tRF-22 in choroidal samples of guinea pigs after 0 (control [Ctrl]), 2, 4, or 8 weeks of FD induction (n = 6 choroid tissues per group). **F** qRT-PCR assays were conducted to detect the expression of tRF-22 in the choroid of C57BL/6 J mice after 0 (Ctrl), 2, 4, or 8 weeks of FD induction (n = 6 choroid tissues per group; *P < 0.05 vs. Ctrl group)

To explore the role of tRF-22 during myopia development, we first detected the expression pattern of tRF-22 in the model of form-deprived myopia (FDM). According to MINTbase v2.0 (http://cm.jefferson.edu/MINTbase/), we found that the length of tRF-22-8BWS72092 (tRF-22) was 22 nts, which is a perfect match to the 3′ end of tRNA^Gln−CTG^. Northern blot analysis validated the expression of tRF-22 (Fig. 1A, B). qRT-PCR assays demonstrated that tRF-22 was highly enriched in choroid tissues and have higher expression levels than that in retinal or scleral tissues (Fig. 1C, D). The myopia of guinea pigs was induced by FD and the fellow eye served as the control. At 2-, 4-, and 8-week after form deprivation, the FD eyes developed relative myopia as shown by refraction error, elongated axial length, and increased vitreous chamber depth compared with the contralateral control eyes (Additional file 2: Fig. S1). The total RNAs were extracted from the choroid tissues. qRT-PCR assays showed that tRF-22 expression was remarkably down-regulated in myopic choroid (Fig. 1E). To verify our findings, we conducted FD treatment in C57BL/6 J mice. Consistent with the above-mentioned results in guinea pigs, qRT-PCR assays indicated that tRF-22 displayed a time-dependent decrease in myopic choroid (Fig. 1F).

### Overexpression of tRF-22 can suppress myopic changes induced by FD treatment

**Fig. 2:**
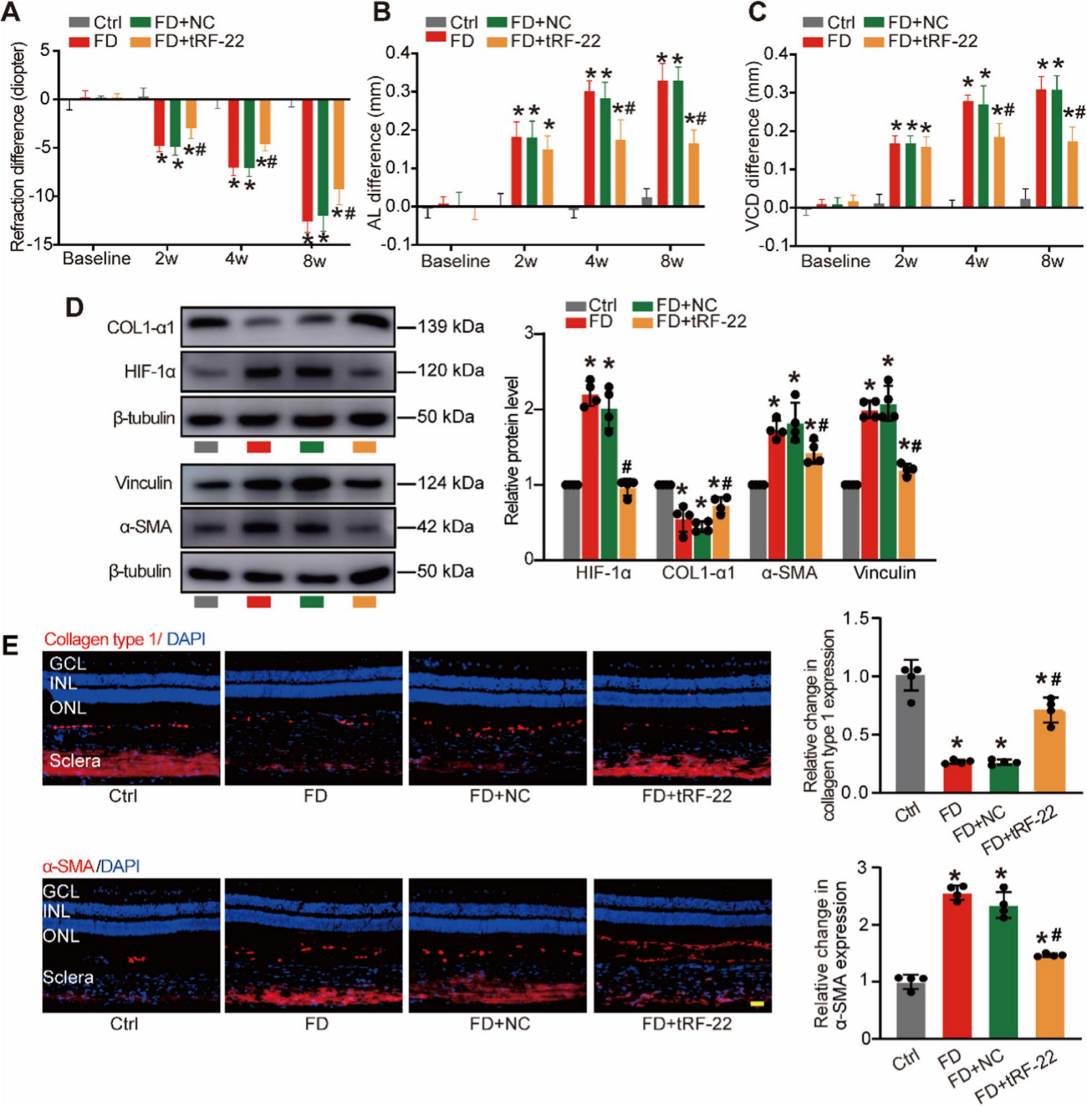
Overexpression of tRF-22 can suppress myopic changes induced by FD treatment. **A**–**C** The FD eyes of guinea pigs received an intravitreal injection of scrambled agomir (NC group), tRF-22 agomir (tRF-22 group), or left untreated (Ctrl group). Interocular differences in refractive errors (**A**), VCD (**B**), and AL (**C**) in guinea pigs was detected to investigate the role of tRF-22 in the progression of myopia (n = 6 *P < 0.05 vs. Ctrl group, ^#^*P* < 0.05 tRF-22 + FD group vs. NC + FD group). **D** Western blots were used to detect the expression of HIF-1α, COL1α1 (fibroblasts marker), α-SMA, and vinculin (myofibroblasts markers) in the sclera after up-regulating tRF-22 (n = 4, *P < 0.05 vs. Ctrl group, ^#^*P* < 0.05 tRF-22 + FD group vs. NC + FD group). **E** Immunofluorescent staining was performed to determine COL1α1 and α-SMA expression in the sclera after up-regulating tRF-22 (n = 4, *P < 0.05 vs. Ctrl group, ^#^*P* < 0.05 tRF-22 + FD group vs. NC + FD group). Scale bars, 100 mm

We next sought to investigate the role of tRF-22 in the progression of myopia. Guinea pigs received intravitreous injections of tRF-22 agomir, Scr agomir, or left untreated. qRT-PCR analysis showed that tRF-22 was obviously increased in choroid tissues after intravitreous injections of tRF-22 agomir (Additional file 2: Fig. S2). Eight weeks after FD treatment, tRF-22 overexpression led to improved refractive errors (Fig. 2A). tRF-22 overexpression also retarded axial length (AL) (Fig. 2B) and vitreous chamber depth (VCD) elongation (Fig. 2C).

Accumulating evidence suggests that hypoxia is a crucial inducer of scleral ECM remodeling during myopia [28]. Since HIF-1α is an indicator of tissue hypoxia, we detected the levels of HIF-1α expression in the sclera after 8-week FD treatment. Western blot analysis showed that tRF-22 overexpression led to reduced expression of HIF-1α (Fig. 2D). During myopia, the accumulation of HIF-1α in scleral microenvironment may trigger myofibroblast transdifferentiation, which is characterized by reduced collagen biosynthesis and increased α-SMA expression [28, 29]. Western blots and immunofluorescence staining assays were conducted to detect the expression of myofibroblast markers (α-SMA and vinculin) and fibroblast marker (COL1α1) in the sclera after 8-week FD treatment. The results revealed that tRF-22 overexpression could reverse the down-regulation of COL1α1 expression and the up-regulation of a-SMA and vinculin expression in the sclera induced by FD treatment (Fig. 2D, E). Thus, tRF-22 overexpression could inhibit myofibroblast transdifferentiation and reverse collagen production and retard the progression of myopia in vivo.

### Overexpression of tRF-22 ameliorates choroidal vascular dysfunction in vivo and ex vivo

**Fig. 3:**
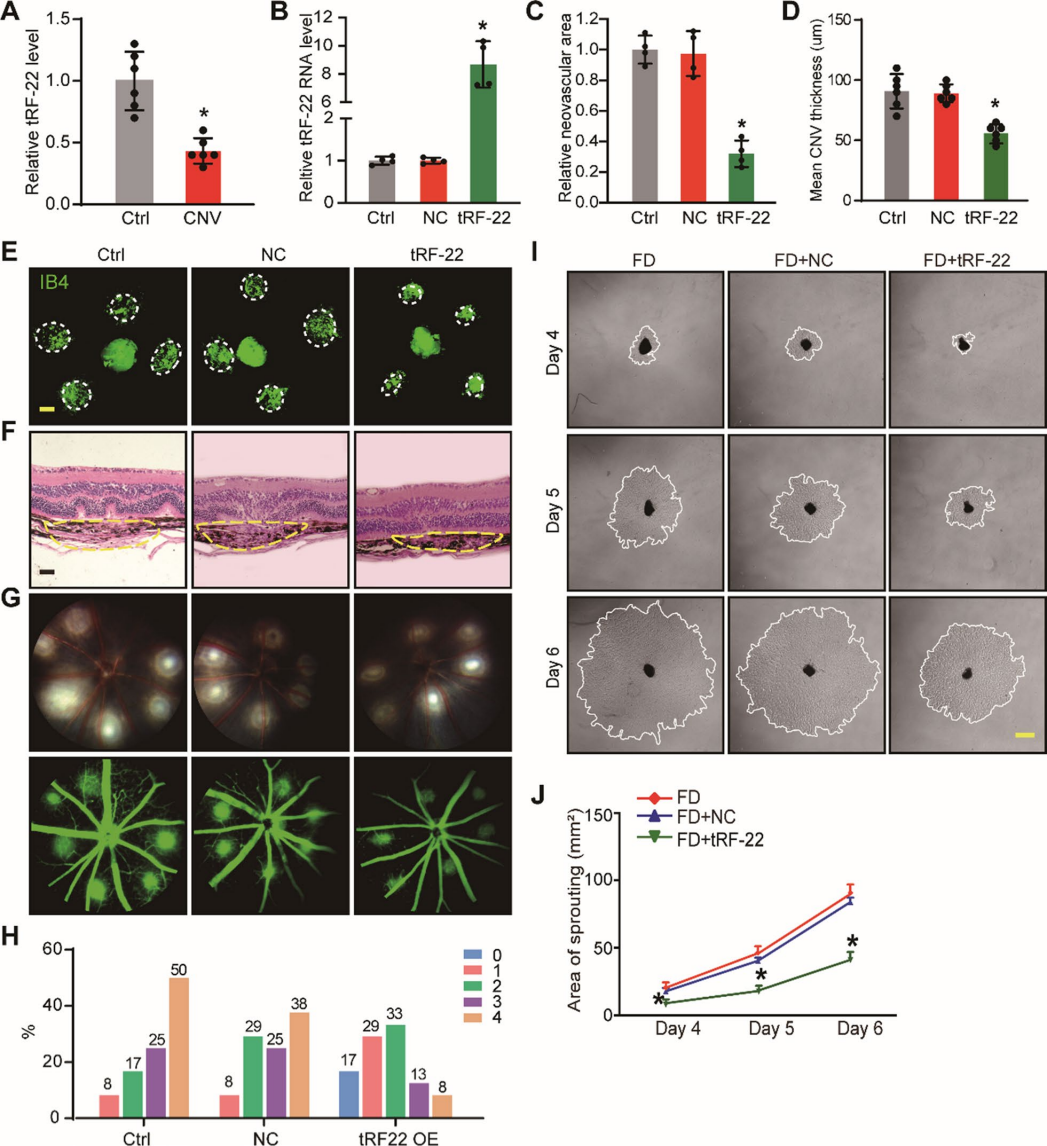
Overexpression of tRF-22 ameliorates choroidal vascular dysfunction in vivo and ex vivo*. ***A** qRT-PCR assays were conducted to detect the expression of tRF-22 in the retina-choroid complex of C57BL/6 J mice after laser injury (n = 6 choroid tissues per group). **B** C57BL/6J mice received an intravitreal injection of scrambled agomir, tRF-22 agomir, or left untreated (Ctrl) for 7 days. qRT-PCR assays were conducted to detect tRF-22 level (n = 4). **C** and **E** At day 14 after CNV induction, IB4 immunofluorescence and quantification were conducted to detect CNV in the flat-mounted choroidal tissues (n = 4, *P < 0.05 vs. Ctrl group). **D** and** F** Representative images of H&E staining showed the thickness of subretinal CNV lesions 14 days after laser injury Yellow dotted lines denote the lesion areas. Scale bar, 100 μm (n = 4, *P < 0.05 vs. Ctrl group). **G** and **H** Representative late phase fundus fluorescein angiography (FFA) images at indicated time points post laser injury. FA Data are expressed as the incidence of CNV angiographic grades of the total laser impacts in each group. **I** and** J** Choroidal sprouting assay was conducted to detect the angiogenic potency of choroidal explants in scrambled agomir + FD group and tRF-22 agomir + FD group. Representative images of choroidal sprouting areas were shown at indicated time points at day 4, day 5, and day 6 after ex vivo incubation. (n = 4, *P < 0.05 vs. Ctrl group). Scale bar, 500 μm

The previous study has revealed that choroidal vasculopathy can lead to scleral hypoxia and alter myopia progression. We then conducted laser-induced CNV model and found that the expression level of tRF-22 decreased in choroidal vasculopathy (Fig. 3A). To investigate the function of tRF-22 further, we examined the effect of tRF-22 over-expression in two different models.

In the laser-induced CNV model, the fundus of C57BL/6 J mice was photocoagulated with or without intravitreal injection of tRF-22 agomir to increase tRF-22 expression in the choroid (Fig. 3B). Quantitative analysis of flat-mounted choroidal tissues labeled with Isolectin B4 (IB4) showed that tRF-22 agomir group had reduced lesion areas and reduced neovascular areas compared with NC agomir group (Fig. 3C, E). The HE staining and fluorescein angiography (FA) assays were conducted to assess the size and permeability of CNV, and the results showed that overexpression tRF-22 reduced choroidal neovascular area, neovascular thickness (Fig. 3D, F) and vascular leakage (Fig. 3G, H).

In the choroidal sprouting ex vivo model, the choroidal explants from FD eyes and contralateral untreated eyes (Ctrl group) were cultured for 6 days. Choroidal endothelial sprouting was observed on day 4, day 5, and day 6. Compared with the Ctrl group, myopia-related stimuli could lead to increased choroidal angiogenic sprouting ability (Additional file 2: Fig. S3). The FD eyes received intraocular injections of tRF-22 agomir. The results showed that tRF-22 agomir led to decreased sprouting ability (Fig. 3I, J), suggesting that tRF-22 plays an anti-angiogenic role in choroidal neovascularization.

### tRF-22 regulates choroidal vascular endothelial cell function in vitro

**Fig. 4:**
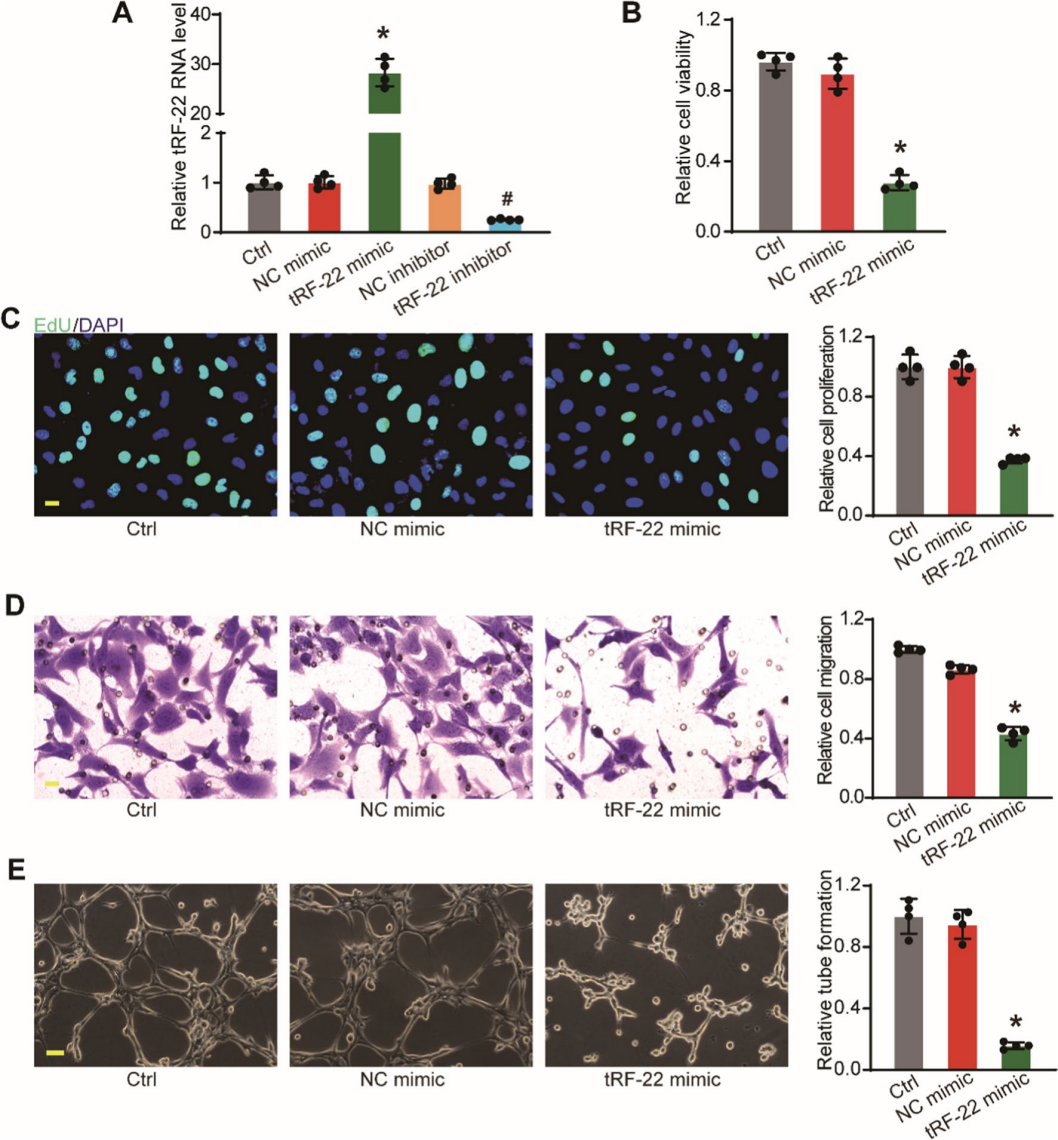
tRF-22 regulates choroidal vascular endothelial cells function in vitro.** A** RF/6A cells were transfected with scrambled mimic (NC mimic group), tRF-22 mimic, scrambled inhibitor (NC inhibitor group), tRF-22 inhibitor, or left untreated (Ctrl group) for 48 h. qRT-PCR assays were conducted to detect tRF-22 levels (n = 4, *P < 0.05 NC mimic group vs. tRF-22 mimic group, ^#^*P* < 0.05 NC inhibitor group vs. tRF-22 inhibitor group). **B** RF/6A cells were transfected with scrambled mimic (NC mimic group), tRF-22 mimic, or left untreated (Ctrl group) for 48 h. Cell viability was detected using MTT assay. (n = 4, *P < 0.05 vs. Ctrl group). **C** Cell proliferation was detected using the EdU detection kit to analyze the incorporation of EdU in DNA synthesis (n = 4, *P < 0.05 vs. Ctrl group). Scale bar: 20 μm. **D** The migration of RF/6A cells was determined using the transwell assay and the cells that migrated through the transwell were quantified (n = 4, *P < 0.05 vs. Ctrl group). Scale bar: 20 μm. **E** RF/6A cells were seeded on the matrigel matrix. The tube-like structures were observed 6 h after cell seeding. The average length of tube formation for each field was statistically analyzed (n = 4, *P < 0.05 vs. Ctrl group). Scale bar: 100 μm

Given the critical role of tRF-22 in governing choroidal vascular functions, we next determined whether tRF-22 played a critical role in choroidal vascular endothelial cell function in vitro. After transfection with tRF-22 mimic, inhibitor and corresponding scrambled control (Scr), we first detected the viability of RF/6A cells under basic conditions. Compared with the Scr group, tRF-22 mimic transfection significantly increased tRF-22 level, by contrast, inhibitor decreased tRF-22 level in RF/6A cells (Fig. 4A). The results of MTT assays showed that tRF-22 overexpression significantly decreased the viability of RF/6A cells (Fig. 4B). we next detected whether tRF-22 is involved in the proliferation, migration, and tube formation capacity of RF/6A cells. EdU assays indicated that tRF-22 overexpression reduced the proliferation ability of RF/6A cells (Fig. 4C). Similarly, the results of Transwell and Matrigel tube formation assays showed that tRF-22 overexpression decreased the migration and tube formation ability of RF/6A cells (Fig. 4D, E). Conversely, down-regulation of tRF-22 led to the increased angiogenic ability of RF/6A cells (Additional file 2: Fig. S4). Under the hypoxic condition, we also revealed that tRF-22 mimic significantly inhibited the viability and angiogenic ability of RF/6A cells. By contrast, tRF-22 down-regulation increased the angiogenic ability of RF/6A cells (Additional file 2: Fig. S5). Collectively, these data suggest that tRF-22 has the anti-angiogenic ability as shown by reduced choroidal endothelial cell proliferation, migration, and tube formation ability in vitro.

### tRF-22 targets METTL3 and regulates its m^6^A methylation activity

**Fig. 5:**
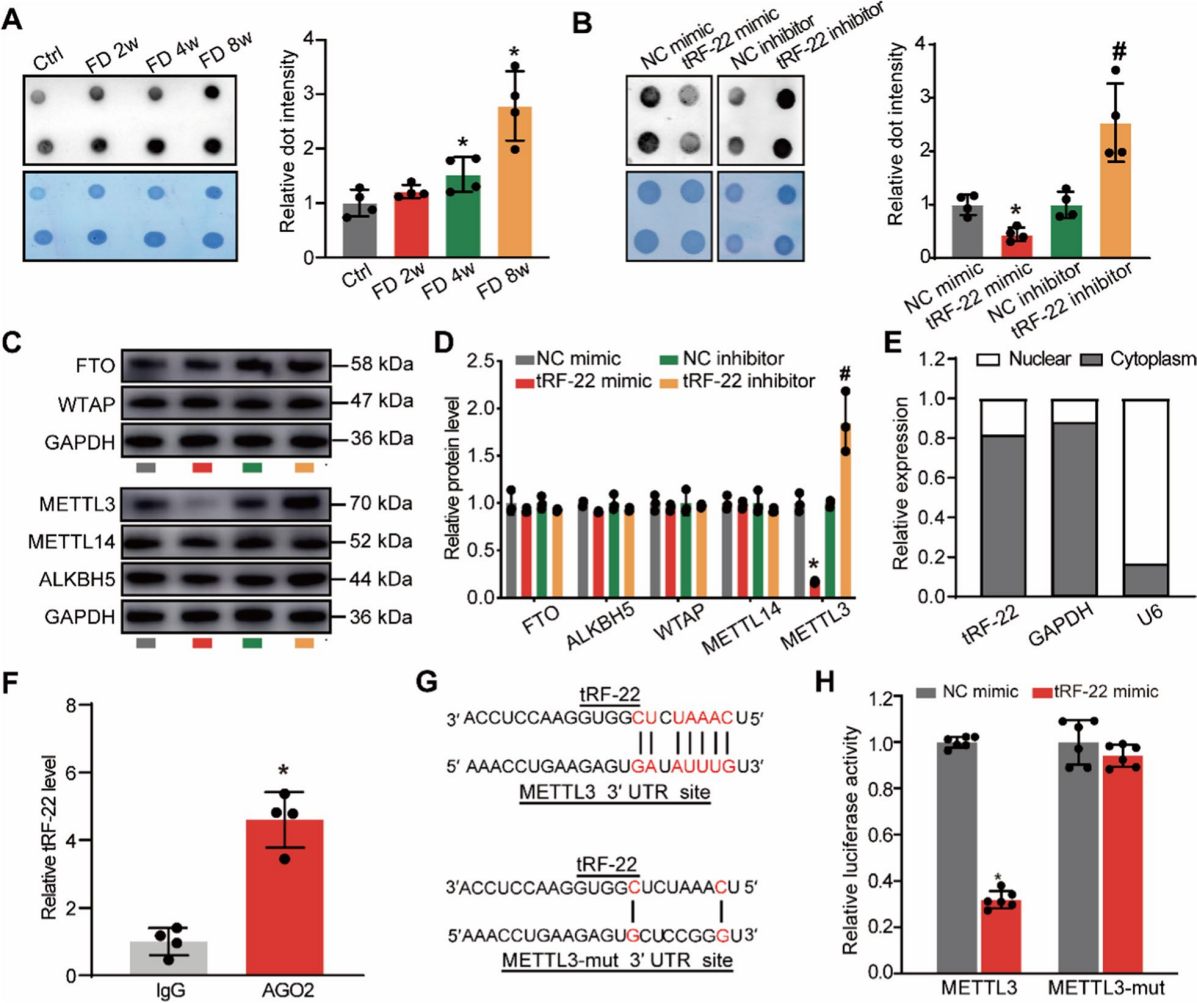
tRF-22 targets METTL3 and regulates its m^6^A methylation activity. **A** The guinea pigs underwent monocular form deprivation (FD) using the translucent eye shield. The levels of m^6^A RNA modification were detected by dot blot assays after 0 (Ctrl), 2, 4, or 8 weeks of FD treatment. (n = 4, *P < 0.05 vs Ctrl group). **B** RF/6A cells were transfected with scrambled mimic (NC mimic group), tRF-22 mimic, scrambled inhibitor (NC inhibitor group), and tRF-22 inhibitor for 48 h. Dot blot assays were conducted to detect the levels of m^6^A RNA modification (n = 4, *P < 0.05 tRF-22 mimic group vs. NC mimic group, ^#^*P* < 0.05 tRF-22 inhibitor group vs. NC inhibitor group). **C** and** D** RF/6A cells were transfected with scrambled mimic (NC mimic group), tRF-22 mimic, scrambled inhibitor (NC inhibitor group), and tRF-22 inhibitor for 48 h. Western blots were conducted to detect the levels of FTO, WTAP, ALKBH5, METTL14, and METTL3 (n = 4, *P < 0.05 tRF-22 mimic group vs. NC mimic group, ^#^*P* < 0.05 tRF-22 inhibitor group vs.NC inhibitor group). **E** The amount of nucleus control transcript (U6), cytoplasm control transcript (GAPDH), and tRF-22 were examined by qRT-PCR assays in the nucleus fractions and cytoplasm fractions of RF/6A cells (n = 4). **F** The cellular fractions were isolated from RF/6As and immunoprecipitated using Ago2 or IgG antibody. The amounts of tRF-22 in the immunoprecipitates were determined by qRT-PCR (n = 4). **G** Schematic representation of the region in 3′-UTR of METTL3 targeted by tRF-22. The mutations introduced into this region are highlighted in gray. **H** Luciferase activity assay was performed in HEK293T cells co-transfected with tRF-22 mimic or Scr mimic and METTL3-WT or METTL3-MUT vector (*P < 0.05 vs. NC mimic group)

Given N^6^-methyladenosine (m^6^A) methylation’s potential involvement in ocular vascular dysfunction [30, 31], we sought to examine whether the levels of m^6^A modification in choroid were altered during myopia development. As shown in Fig. 5A, the levels of m^6^A modification were increased after FDM modeling, indicating a potential interaction between tRF-22 and m^6^A modification. tRF-22 overexpression decreased m^6^A methylation levels, while tRF-22 inhibitor increased m^6^A methylation levels in RF/6A cells (Fig. 5B). Further experiments showed that tRF-22 did not affect the expression levels of m^6^A RNA demethylases (FTO and ALKBH5) or methylases (METTL14, and WTAP) except for methyltransferase-like 3 (METTL3). Western blot assays further verified that the protein levels of METTL3 were decreased after tRF-22 overexpression but increased after tRF-22 inhibition in choroidal endothelial cells (Fig. 5C, D). Currently, several studies about 3′-CCA tRFs revealed that these small RNAs down-regulate gene expression in an RNAi-like mechanism [32, 33]. We next detected the location of tRF-22 in choroidal endothelial cells and subsequently interrogated whether this small RNA could interact with Ago2, the argonaute family member that binds miRNA complexes to target mRNA transcripts. The qRT-PCR analysis revealed that tRF-22 was mainly expressed in the cytoplasm (Fig. 5E). The results of RNA immunoprecipitation (RIP) showed that tRF-22 could be immunoprecipitated by anti-Ago2 (Fig. 5F). These data demonstrated that tRF-22 can repress target mRNAs in an Ago2-dependent, miRNA-like fashion. Bioinformatic tools (MINTbase v2.0, miRanda and Targetscan) predicted that tRF-22 can target the 3′ UTR of METTL3 mRNA (Fig. 5G). To validate if tRF-22 can regulate METTL3 expression by the complementary binding with METTL3 mRNA, we subsequently cloned the 3′ UTR of METTL3 containing the wild-type or -mutated tRF-22 binding site into the luciferase reporter vector (Fig. 5G). Luciferase reporter assays showed that tRF-22 overexpression decreased the luciferase activities of wild-type reporter, while the luciferase activities of mutated vector without tRF-22 could not be unaltered (Fig. 5H). These results suggest that tRF-22 can directly bind to the 3′ UTR region of METTL3 mRNA and down-regulate METTL3 expression.

### METTL3 suppresses Axin1 and Arid1b expression via YTHDF2-mediated mRNA decay

**Fig. 6:**
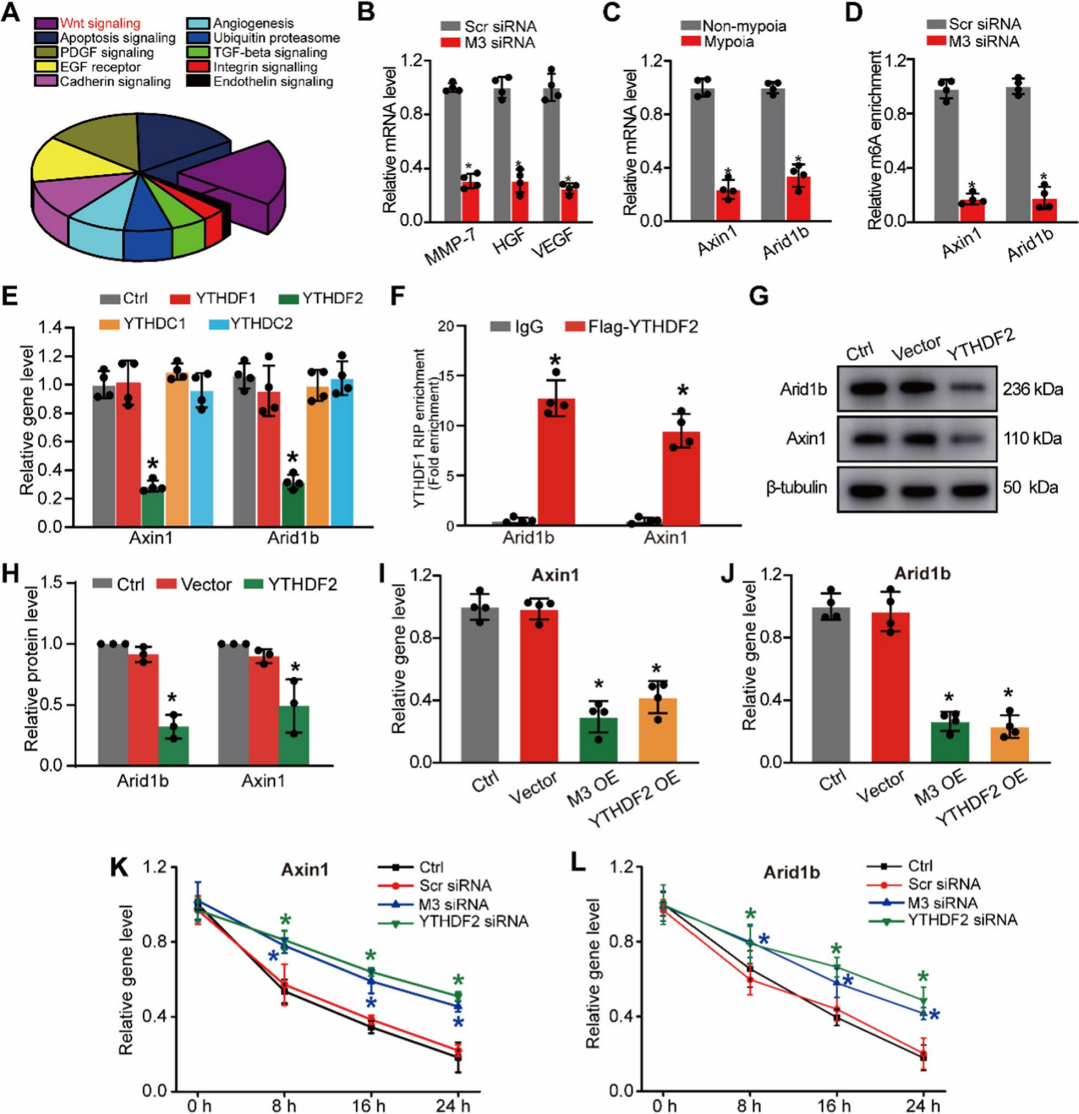
METTL3 suppresses Axin1 and Arid1b expression via YTHDF2-mediated mRNA decay.** A** KEGG pathway analysis was conducted to predict the signaling pathways potentially regulated by myopia. **B** RF/6A cells were transfected with METTL3 (M3) siRNA or scrambled (Scr) siRNA for 24 h. qRT-PCR assays were conducted to detect the levels of MMP-7, HGF, and VEGF (n = 4, *P < 0.05 vs. Scr siRNA group). **C** qRT-PCR assays were conducted to detect the levels of Axin1 and Arid1 in myopic choroid and non-myopic choroid (n = 4, *P < 0.05 vs. Non-myopia group). **D** Gene-specific m^6^A-qPCR assays were conducted to detect the change of m^6^A modification in the specific regions of Axin1 and Arid1 upon METTL3 knockdown in RF/6A cells (n = 4, *P < 0.05 vs. Scr siRNA group). **E** RF/6A cells were transfected with YTHDF1-Flag, YTHDF2-Flag, YTHDC1-Flag, YTHDC2-Flag, or left untreated (Ctrl) for 24 h. qRT-PCR assays were conducted to detect the levels of Axin1 and Arid1b (n = 4, *P < 0.05 vs. Ctrl group). **F** RIP analysis of the interaction of Axin1 and Arid1b in RF/6A cells transfected with YTHDF2-Flag plasmid. Enrichment of Axin1 and Arid1b was determined by qRT-PCR assays and normalized to the input (n = 4, *P < 0.05 vs. IgG group). **G**, **H** Cells were transfected with pcDNA3.1-YTHDF2 (YTHDF2), pcDNA3.1 vector (Vector), or were left untreated (Ctrl) for 24 h. Western blots were used to examineAxin1 and Arid1b expression (n = 3). **I**, **J** RF/6A cells were transfected with pcDNA 3.1 vector, pcDNA 3.1-METTL3, pcDNA 3.1-YTHDF2, or left untreated (Ctrl) for 24 h. qRT-PCR assays were conducted to detect the levels of Axin1 **I** and Arid1b **J** in RF/6A cells (n = 4, *P < 0.05 vs. Ctrl group). **K**, **L** RF/6A cells were transfected with scrambled (Scr) siRNA, METTL3 (M3) siRNA or YTHDF2 siRNA or left untreated (Ctrl) for 12 h, and then exposed to the transcription inhibitor Actinomycin D (5 mg/ml) for 8 h, 16 h, or 24 h. qRT-PCR assays were conducted to detect the levels of Axin1 **K** and Arid1b **L** (n = 4, *P < 0.05 vs. Ctrl group)

To further identify the downstream molecule involved in choroidal vascular dysfunction, we performed RNA sequencing (RNA-seq) assays to detect gene expression patterns between myopic and non-myopic choroidal tissues. We found that 2842 genes were differentially expressed genes between myopic tissues and non-myopic controls, including 1667 up-regulated genes and 1175 down-regulated genes (Additional file 3: Table S2). Meanwhile, these differentially expressed genes were substantially enriched in 10 signaling pathways, including Wnt signaling, Apoptosis signaling, platelet-derived growth factor (PDGF) signaling, epidermal growth factor receptor (EGFR) signaling, Cadherin signaling, Angiogenesis, Ubiquitin proteasome pathway, TGF-beta signaling, Integrin signaling, and Endothelin signaling. Among these signaling pathways, Wnt signaling pathway was the most enriched one that was potentially affected by myopia and potentially regulated by METTL3 (Fig. 6A).

We next determined the association between METTL3 expression and Wnt signaling activation. qRT-PCR assays showed that METTL3 knockdown decreased the expression of VEGF, MMP-7 and HGF, which are the downstream members of Wnt signaling (Fig. 6B). Axin1 and Arid1b are the regulators of Wnt signaling [32, 34]. They are differentially expressed between myopic and non-myopic eyes, which were verified by RNA-seq and qRT-PCR assays (Fig. 6C and Additional file 3: Table S2). To verify whether METTL3 regulated the methylation status of Axin1 and Arid1b in choroidal endothelial cells, we first predicted the m^6^A methylation sites of Axin1 and Arid1b with the SRAMP program and the results showed that the mRNA of Axin1 and Arid1b carrying more than 10 potential m^6^A methylation sites with the high confidence (Additional file 2: Figure S6). Gene-specific m^6^A-qPCR assays were conducted to further determine m^6^A abundance of Axin1 and Arid1b after METTL3 intervention. METTL3 knockdown significantly reduced the m^6^A abundance of Axin1 and Arid1b (Fig. 6D), suggesting that Axin1 and Arid1b are the direct targets of METTL3.

It is known that m^6^A exerts its biological roles via the specific m^6^A-binding proteins, including YTHDF1-2 and YTH domain containing (YTHDC) proteins 1–2, which can regulate the downstream proteins via regulating mRNA stability and translation [35, 36]. We thus detected the underlying mechanism by which m^6^A modification regulated the stability and translation of Axin1 and Arid1b in RF/6A cells. As shown in Fig. 6E, overexpression of YTHDF2, but not YTHDF1 or YTHDC1-2 significantly reduced the levels of Axin1 and Arid1b RIP-qPCR analysis indicated that Axin1 and Arid1b were the direct targets of YTHDF2 (Fig. 6F). Overexpression of YTHDF2 reduced expression of Axin1 and Arid1b in RF/6As (Fig. 6G, H). The levels of Axin1 and Arid1b expression were obviously decreased in RF/6A cells after YTHDF2 overexpression, suggesting that YTHDF2 recognition of m^6^A may lead to mRNA decay of Axin1 and Arid1b mRNA (Fig. 6I, J). To determine whether YTHDF2 regulated Axin1 and Arid1b mRNA through mRNA decay mechanism, we detected the levels of Axin1 and Arid1b following Actinomycin D treatment. Compared with the control group, the remaining levels of Axin1 and Arid1b were significantly increased in RF/6A cells after METTL3 knockdown or YTHDF2 knockdown (Fig. 6K, L).

### tRF-22 regulates m^6^A methylation of Axin1/Arid1b mRNA via METTL3

**Fig. 7:**
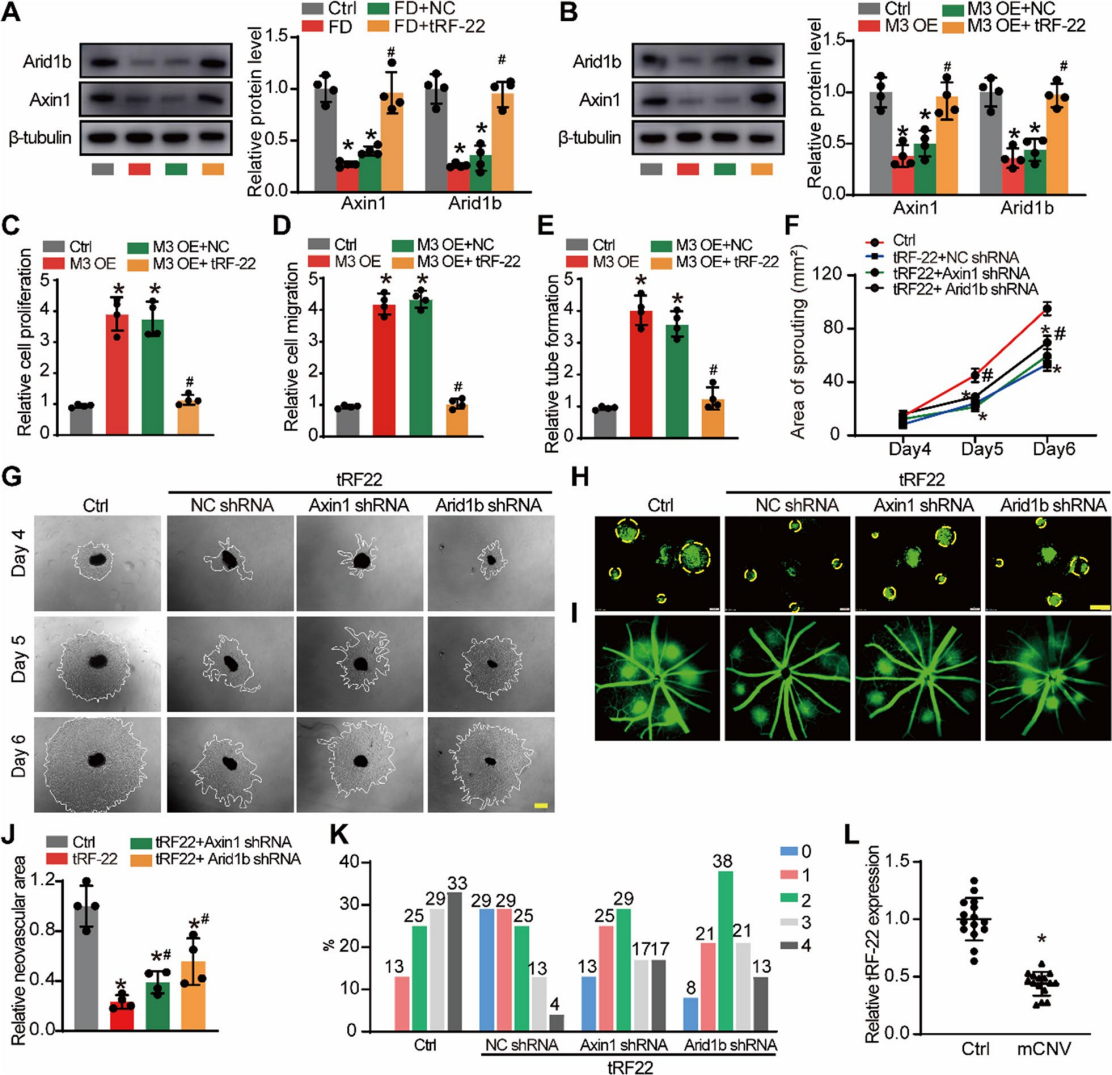
tRF-22 regulates m^6^A methylation of Axin1/Arid1b mRNA via METTL3.** A** The eyes of C57BL/6 J mice received intravitreal injections of Scr agomir, tRF-22 agomir, or were left untreated (Ctrl) for 2 months. Western blots were conducted to detect the levels of Axin 1 and Arid1b after FD treatment. (n = 4, *P < 0.05 vs. Ctrl group, ^#^*P* < 0.05 Scr agomir group vs. tRF-22 agomir group). **B** RF/6A cells were transfected with pcDNA 3.1 vector, pcDNA 3.1-METTL3, pcDNA 3.1-METTL3 + Scr mimic or pcDNA 3.1-METTL3 + tRF-22 mimic for 24 h. Western blots were conducted to detect the levels of Axin1 and Arid1 (n = 4, *P < 0.05 vs. Ctrl group, ^#^*P* < 0.05 M3 OE + tRF-22 mimic group vs. M3 OE + Scr mimic group). **C** EdU assays were conducted to detect the proliferation of RF/6A cells (n = 4). **D** Transwell assays were conducted to detect the migration of RF/6A cells (n = 4). **E** Matrigel assays were conducted to detect the tube formation of RF/6A cells (n = 4, *P < 0.05 vs. Ctrl group, ^#^*P* < 0.05 M3 OE + tRF-22 mimic group vs. M3 OE + Scr mimic group). **F**, **G** Choroidal sprouting assay was conducted to detect the angiogenic potency of choroidal explants in FD group (Ctrl), scrambled shRNA + tRF-22 agomir FD group (tRF-22 + NC shRNA group), tRF-22 agomir + Axin 1 shRNA FD group (tRF-22 + Axin1 shRNA group) and tRF-22 agomir + Arid1b shRNA FD group (tRF-22 + Arid1b shRNA group). Representative images of choroidal sprouting areas were shown at indicated time points at day 4, day 5, and day 6 after ex vivo incubation. Scale bar, 500 μm. (n = 4, (n = 4, *P < 0.05 vs. Ctrl group, ^#^*P* < 0.05 vs. tRF-22 + NC shRNA). **H**, **J** At day 14 after CNV induction, IB4 immunofluorescence and quantification were conducted to detect CNV in the flat-mounted choroidal tissues (n = 4). **I**, **K** Representative late phase fundus fluorescein angiography (FFA) images at indicated time points post laser injury. FA Data are expressed as the incidence of CNV angiographic grades of the total laser impacts in each group. **L** Aqueous humor (AH) was obtained from the patients with myopic CNV (mCNV) (n = 15 eyes) and the patients with cataract (Ctrl, n = 15 eyes). qRT-PCR assays were conducted to detect the expression of tRF-22. *P < 0.05 vs. Ctrl group

Given that Axin1 and Arid1b were the downstream targets of METTL3-induced m^6^A modification, we next sought to investigate the role of tRF-22/METTL3/Axin1/Arid1b axis in choroid endothelial function. As shown in Fig. 7A, the levels of Axin1 and Arid1b expression were decreased in the FD group. Injection of tRF-22 agomir could partially reverse the reduced expression of Axin1 and Arid1b. In RF/6A cells, METTL3 overexpression lead to reduced expression of Axin1 and Arid1b, while tRF-22 mimic overexpression could partially reverse METTL3-mediated reduction of Axin1 and Arid1b (Fig. 7B).

We next examined the role of tRF-22/METTL3 signaling axis in choroidal endothelial cell function in vitro. METTL3 overexpression increased the proliferation, migration, and tube formation ability in RF/6A cells. tRF-22 mimic could alleviate the effects of METTL3 overexpression on endothelial angiogenic functions as shown by decreased the proliferation, migration, and tube formation ability compared with METTL3 overexpression group (Fig. 7C–E). To further validate that tRF-22 regulates choroidal vasculopathy via the METTL3/Axin1/Arid1b axis in vivo, we conducted rescue experiments by co-infecting tRF-22 agomir and Axin1/Arid1b shRNA in FD model and laser-induced CNV model. The choroidal tissues from FD mice were incubated onto Matrigel to induce choroidal vascular sprouting. tRF-22 overexpression reduced area of choroidal sprouting, while Axin1 or Arid1b silencing could partially inhibit the effects of tRF-22 overexpression (Fig. 7F, G). Consistently, in laser-induced CNV model, the effect of tRF-22 on ameliorating choroidal vasculopathy was attenuated by inhibiting Axin1 or Arid1b expression (Fig. 7H–K).

To reveal the clinical relevance of tRF-22-mediated signaling, we examined the expression levels of tRF-22 in the patients with myopic CNV and the patients with cataract. qRT-PCRs showed that the levels of tRF-22 were markedly reduced in the aqueous humor of the patients with mCNV (Fig. 7L).

## Discussion

Increasingly studies have identified the wide expression of tRFs in multiple cell types and external stimuli could lead to altered expression of tRFs [16]. In this study, we identified that tRF-22, derived from the 3′ ends of tRNA^Gln−CTG^, was shown as a key regulator of choroidal vascular function involved in the progression of myopia. The levels of tRF-22 expression were significantly decreased in the myopic eyes. Up-regulating tRF-22 level could alleviate choroid vascular dysfunction reduce scleral hypoxia, suppress scleral ECM remodeling, and ultimately retard the progression of myopia. Mechanistically, tRF-22 could suppress the m^6^A modification of Axin1 and Arid1b mRNA by decreasing METTL3 expression via blocking its RNA methylation activity.

Choroid, a highly vascularized tissue located between the sclera and retina, plays a vital role in the transmission of signaling molecules from retina to sclera [8, 38]. Current clinical evidence reveals that choroid thickness decreases during the progression of myopia. By contrast, the macular retinal thickness remains unchanged or slightly thickened, implicating that choroidal change may precede the retina during myopia and be a possible trigger to induce myopia [12, 39]. In this study, we revealed a novel mechanism of tRF-22 involvement in choroid vascular dysfunction. Decreased tRF-22 could aggravate choroid vascular dysfunction, causing the lack of oxygen and nutrients for retina and sclera. Under the hypoxic condition, VEGF expression is induced through the activation of HIF-1α signaling and activates choroid endothelial cells to initiate myopic CNV, which could accelerate the progression of myopia [29, 40]. Overexpressing tRF-22 could reduce endothelial angiogenic effects and suppress myopic CNV as shown both in laser-induced CNV model and choroidal sprouting ex vivo model. Previous studies have reported that hypoxia is a key regulator that promotes the sclera transdifferentiation toward myofibroblast and ECM remodeling [29, 41]. Increased tRF-22 could protect choroidal vascular function and repress hypoxia-induced scleral ECM remodeling, which in turn played a role in alleviating myopia progression.

Mounting evidence has revealed that tRFs regulate a variety of biological processes, including gene expression, ribosome biogenesis, stress granule assembly, translation initiation and elongation [42]. Among them, especially 3′-tRFs, show its miRNA-like role in gene expression regulation by directly binding to the 3′-UTR region [33]. Interestingly, we found that tRF-22 was derived from the 3′ end of a mature tRNA and had similar structural and functional characteristics of miRNAs. Based on these findings, we speculated that tRF-22 regulated choroidal dysfunction in a miRNA-like mechanism. We showed that tRF-22 could directly bind to the 3′-UTR region of METTL3 mRNA and repressed its translation. METTL3, the main methyltransferase, is critical for the m^6^A modification. The role of m^6^A modification in vascular function has gradually been recognized. For instance, during endothelial-to-hematopoietic transition, m^6^A modification can determine cell fate to specify the earliest hematopoietic stem/progenitor cells [43]. Silencing of m^6^A reader YTHDF2 can provoke inflammation, vascular reconstruction, and metastatic progression in hepatocellular carcinoma [44]. Knockdown of m^6^A writer METTL3 can reduce pathological neovascularization in both oxygen-induced retinopathy model and corneal alkali burn model [28]. We showed that METTL3 knockdown abrogated the effect of tRF-22 on endothelial cell viability, proliferation, migration, and tube formation ability. Thus, tRF-22 regulates choroidal vascular function via repressing METTL3-mediated m^6^A modification.

RNA-Seq analysis has revealed that Wnt signaling in choroid is potentially involved in the progression of myopia. A recent study has demonstrated that overactivation of Wnt signaling drives myopia development [45], and increasing studies suggest that Wnt signaling has emerged as a key regulator of ocular angiogenesis during ocular development and ocular vascular diseases [46]. Axin1 is a concentration-limiting component of β-catenin destruction complex, which can negatively regulate the Wnt signaling pathway [34]. Arid1b is a chromatin remodeling factor that can bind to β-catenin and inhibit Wnt/β-catenin-mediated genes’ transcription through BAF core subunit BRG1 [35]. We found that the expression levels of Axin1 and Arid1b were remarkably decreased in myopic choroid tissues. m^6^A abundance of Axin1 and Arid1b was markedly decreased upon METTL3 knockdown. YTHDF2 binding could lead to mRNA decay of Axin1 and Arid1b, which could release the inhibitory effects of Axin1 and Arid1b on Wnt signaling. Moreover, tRF-22 blocked METTL3-mediated m^6^A methylation of Axin1 and Arid1b mRNA transcripts, which led to increased expression of Axin1 and Arid1b. Functional studies revealed that overexpression of Axin1 or Arid1b could exert inhibitory effects on endothelial angiogenic abilities of RF/6A cells. By contrast, tRF-22 inhibition interrupted the inhibitory effects of Axin1 or Arid1b on endothelial angiogenic effects.

Although we provide advances of tRFs in myopia, there are several limitations of our study. First, the naming of tRFs still lacks unified rules, which poses numerous inconveniences in research. In our study, we named the tRF as tRF-22 based on its length, but others may not name it that way. Second, tRFs exhibit similarity in their sequences and lengths, making it challenging to establish standardized methodologies for their detection and characterization. Overcoming these limitations will require concerted efforts in developing robust methodologies and conducting comprehensive functional studies in relevant biological contexts.

## Conclusion

Taken together, our study reveals a protective role of tRF-22 in the progression of myopia via regulating choroidal vascular function. Mechanistically, tRF-22 acts as an anti-angiogenic factor by blocking METTL3-mediated m^6^A methylation of Axin1 or Arid1b mRNA transcripts and releasing the inhibitory effect of Axin1 or Arid1b on Wnt signaling. Thus, the therapeutic strategy that targets tRF-22/METTL3 axis in the choroid is critical for the management and prevention of myopic pathology.

## Supplementary Information

**Additional file 1: Table S1.** Primers for qRT-PCR.

**Additional file 2: Figure S1.** Establishment of the myopia model. **Figure S2.** tRF-22 overexpression retards the progression of myopia in vivo. **Figure S3.** tRF-22 regulates choroidal vascular dysfunction in vivo* and *ex vivo. **Figure S4.** tRF-22 down-regulation affects choroidal endothelial cell function in vitro. **Figure S5.** tRF-22 regulates choroidal endothelial cell function in vitro under hypoxic conditions. **Figure S6.** Prediction of m^6^A methylation sites of Axin1 and Arid1b using the SRAMP program.

**Additional file 3: Table S2.** The results of RNA sequencingassays.

## Data Availability

All data generated or analysed during this study are included in this published article.

## Abbreviations

ECM: Scleral extracellular matrix
tRFs: Transfer RNA-derived fragments
CNV: Choroidal neovascularization
FDM: Form-deprived myopia
VCD: Vitreous chamber depth
AL: Axial length
FA: Fluorescein angiography
RIP: RNA immunoprecipitation
m^6^A: N^6^-methyladenosine
METTL3: Methyltransferase-like 3
RNA-seq: RNA sequencing
PDGF: Platelet-derived growth factor
EGFR: Epidermal growth factor receptor
YTHDC: YTH domain containing
