# Supplementary material for: Targeting choroidal vasculopathy via up-regulation of tRNA-derived fragment tRF-22 expression for controlling progression of myopia
Source: J Transl Med. 2023 Jun 24;21:412. doi: 10.1186/s12967-023-04274-5 (PMC10290315; doi:10.1186/s12967-023-04274-5)
Supplement: Supplementary file 1 — Additional file 1: Table S1. Primers for qRT-PCR. [file 12967_2023_4274_MOESM1_ESM.docx]

**Additional file 1: Table S1：Primers for qRT-PCR**

| **Primers** | **Sequence (5’-3’)** |
| --- | --- |
| Axin1 Forward | ATGCAGAGTCCCAAAATGAATGT |
| Axin1 Reverse | GGGGCATCTTCGGTGAAACTT |
| Arid1 Forward | CTCCCCTGCGAGTATTCCAG |
| Arid1 Reverse | TGCCTGTCATAAAACCTCTTTCC |
| β-actin Forward | TTGTTACAGGAAGTCCCTTGCC |
| β-actin Reverse | ATGCTATCACCTCCCCTGTGTG |
| MMP-7 Forward | ATGTGGAGTGCCAGATGTTGC |
| MMP-7 Reverse | AGCAGTTCCCCATACAACTTTC |
| HGF Forward | GCTATCGGGGTAAAGACCTACA |
| HGF Reverse | CGTAGCGTACCTCTGGATTGC |
| VEGF Forward | AGGGCAGAATCATCACGAAGT |
| VEGF Reverse | AGGGTCTCGATTGGATGGCA |
