# Supplementary material for: Targeting choroidal vasculopathy via up-regulation of tRNA-derived fragment tRF-22 expression for controlling progression of myopia
Source: J Transl Med. 2023 Jun 24;21:412. doi: 10.1186/s12967-023-04274-5 (PMC10290315; doi:10.1186/s12967-023-04274-5)
Supplement: Supplementary file 2 — Additional file 2: Figure S1. Establishment of the myopia model. Figure S2. tRF-22 overexpression retards the progression of myopia in vivo. Figure S3. tRF-22 regulates choroidal vascular dysfunction in vivo and ex vivo. Figure S4. tRF-22 down-regulation affects choroidal endothelial cell function in vitro. Figure S5. tRF-22 regulates choroidal endothelial cell function in vitro under hypoxic conditions. Figure S6. Prediction of m6A methylation sites of Axin1 and Arid1b using the SRAMP program. [file 12967_2023_4274_MOESM2_ESM.docx]

**Additional file 2**

**
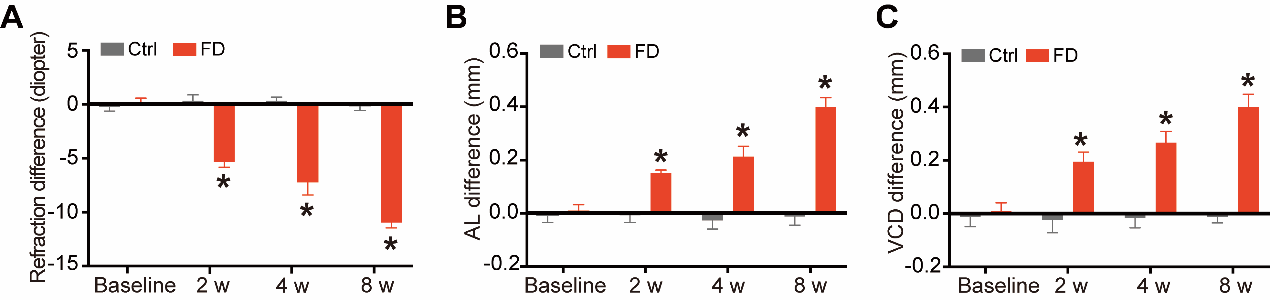
**

**Figure S1: Establishment of the myopia model.** **(A-C)** The three-week-old guinea pigs underwent monocular FD using a translucent eye shield. Three ocular biometric parameters, including refractive state (A), axial length (B, AL), and vitreous chamber depth (C, VCD), were used to estimate the establishment of myopia model (n = 6 eyeballs per group).

**
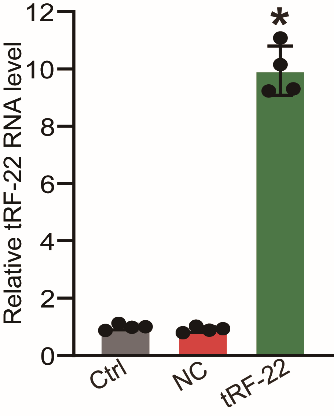
**

**Figure S2: tRF-22 overexpression retards the progression of myopia *in vivo.*** The FD eyes of guinea pigs received an intravitreal injection of scrambled agomir (NC), tRF-22 agomir (tRF-22), or left untreated (Ctrl). qRT-PCR assays were conducted to detect tRF-22 levels (n = 4; **P* < 0.05 versus Ctrl group).

**
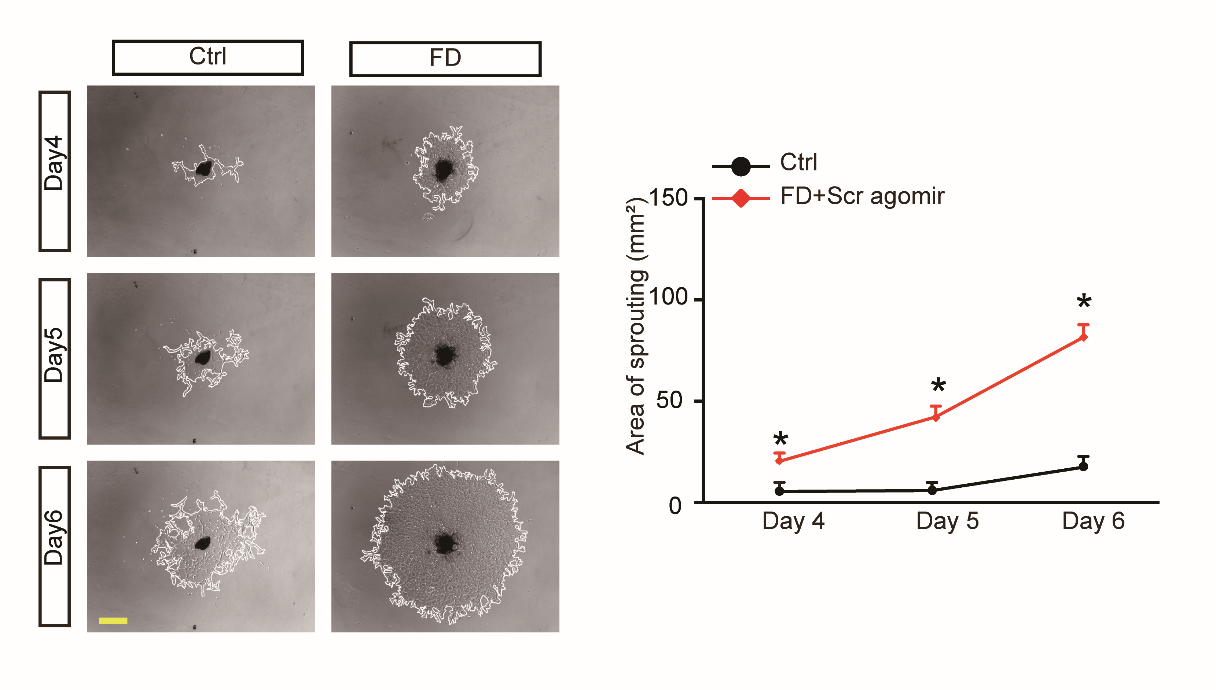
**

**Figure S3: tRF-22 regulates choroidal vascular dysfunction *in vivo* and *ex vivo.*** Choroidal sprouting assay was conducted to compare the angiogenic potency of choroidal explants between FD group and untreated group (Ctrl group). Representative images of choroidal sprouting areas were shown at indicated time points at day 4, day 5, and day 6 after *ex vivo* incubation. (n = 4, **P* < 0.05 vs. Ctrl group). Scale bar, 500 μm.

**
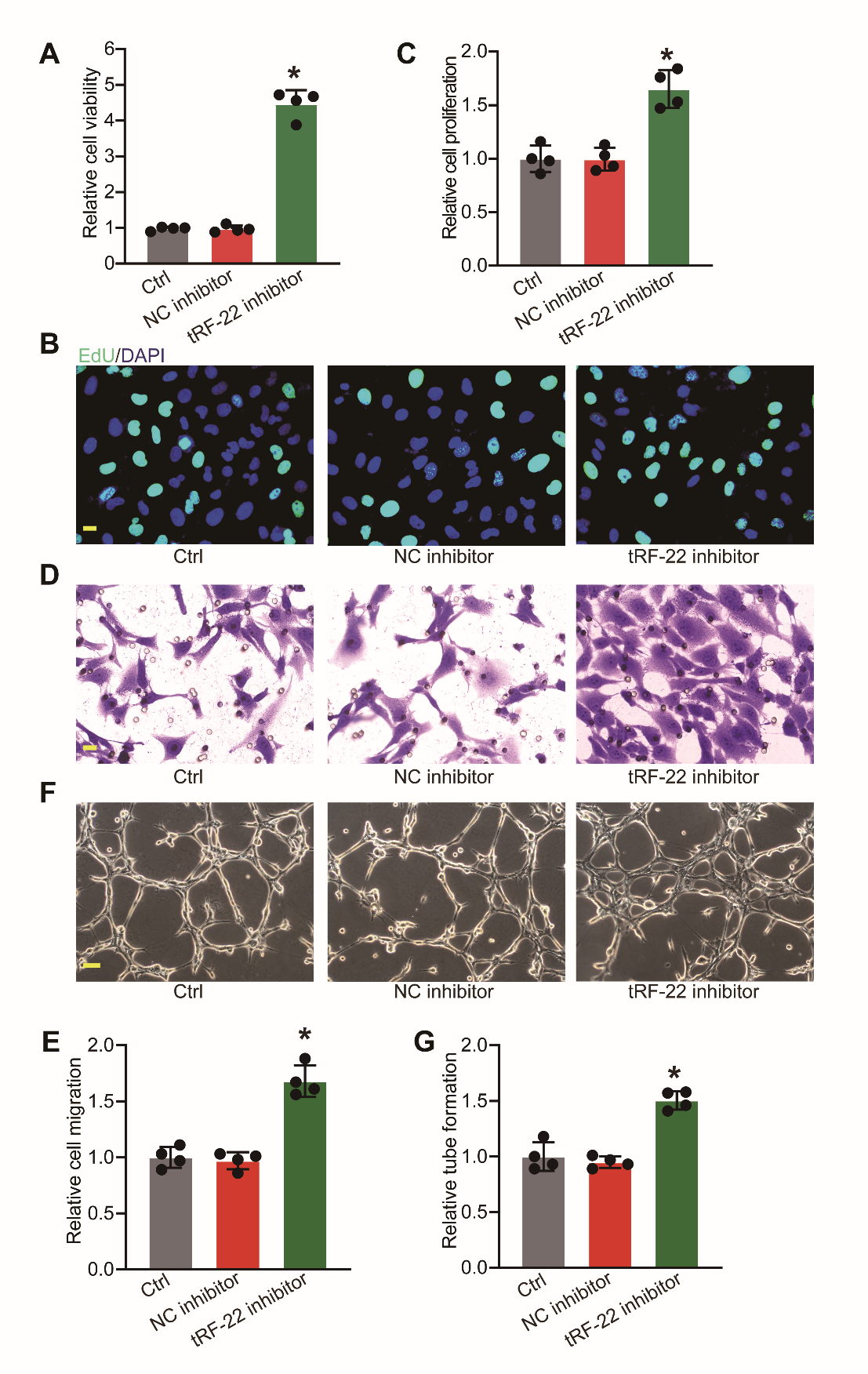
**

**Figure S4: tRF-22 down-regulation affects choroidal endothelial cell function *in vitro.*** **(A)** RF/6A cells were transfected with Scr inhibitor (NC inhibitor), tRF-22 inhibitor or left untreated (Ctrl) for 48 h. Cell viability was detected using MTT assay (n = 4, **P* < 0.05 vs. Ctrl group). **(B and C)** Cell proliferation was detected using EdU detection kit (n = 4, **P* < 0.05 vs. Ctrl group). Scale bar: 20 μm. **(D and E)** The migration of RF/6A cells was detected using Transwell assay and the cells that migrated through the Transwell were quantified (n = 4, **P* < 0.05 vs. Ctrl group). Scale bar: 20 μm. RF/6A cells were seeded on the matrigel matrix. The tube-like structures were observed 6 h after cell seeding. **(F and G)** The average length of tube formation for each field was statistically analyzed (n = 4, **P* < 0.05 vs. Ctrl group). Scale bar: 100 μm.

**
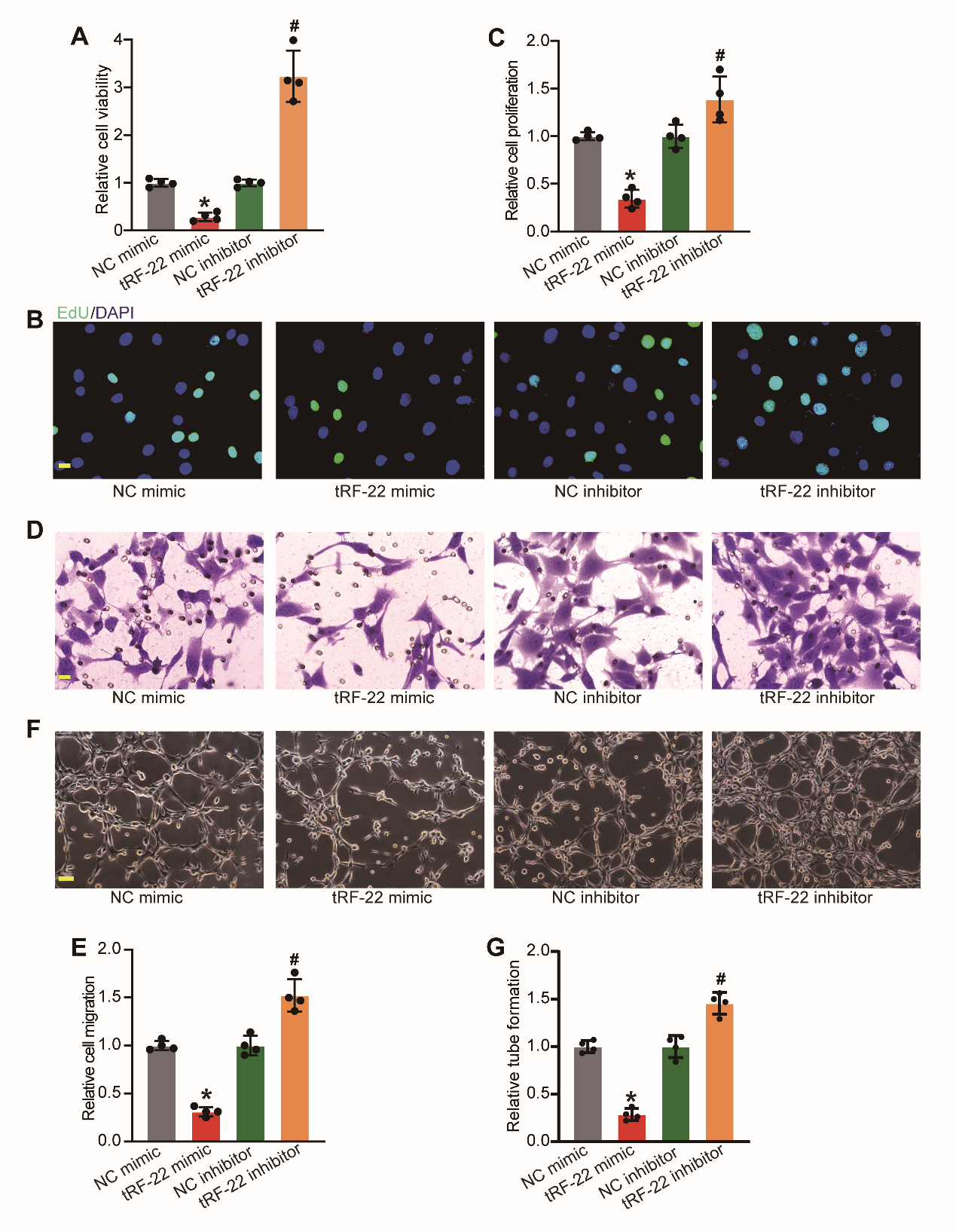
Figure S5: tRF-22 regulates choroidal endothelial cell function *in vitro* under hypoxic conditions. (A)** RF/6A cells were transfected with Scr inhibitor, tRF-22 inhibitor, Scr mimic, or tRF-22 mimic, and then exposed to hypoxic condition (1% of oxygen) for 48 h. Cell viability was detected using MTT assay (n = 4, **P* < 0.05 vs. NC mimic group, ^#^*P*<0.05 vs. NC inhibitor group). **(B and C)** Cell proliferation was detected using EdU detection kit (n = 4, **P* < 0.05 vs. Ctrl group, ^#^*P*<0.05 vs. NC inhibitor group). Scale bar: 20 μm. **(D and E)** The migration of RF/6A cells was detected using Transwell assay and the cells that migrated through the Transwell were quantified n = 4, **P* < 0.05 vs. Ctrl group, ^#^*P*<0.05 vs. NC inhibitor group). Scale bar: 20 μm. **(F and G)** RF/6A cells were seeded on the matrigel matrix. The tube-like structures were observed 6 h after cell seeding. The average length of tube formation for each field was statistically analyzed (n = 4, **P* < 0.05 vs. Ctrl group, ^#^*P*<0.05 vs. NC inhibitor group). Scale bar: 100 μm.

**
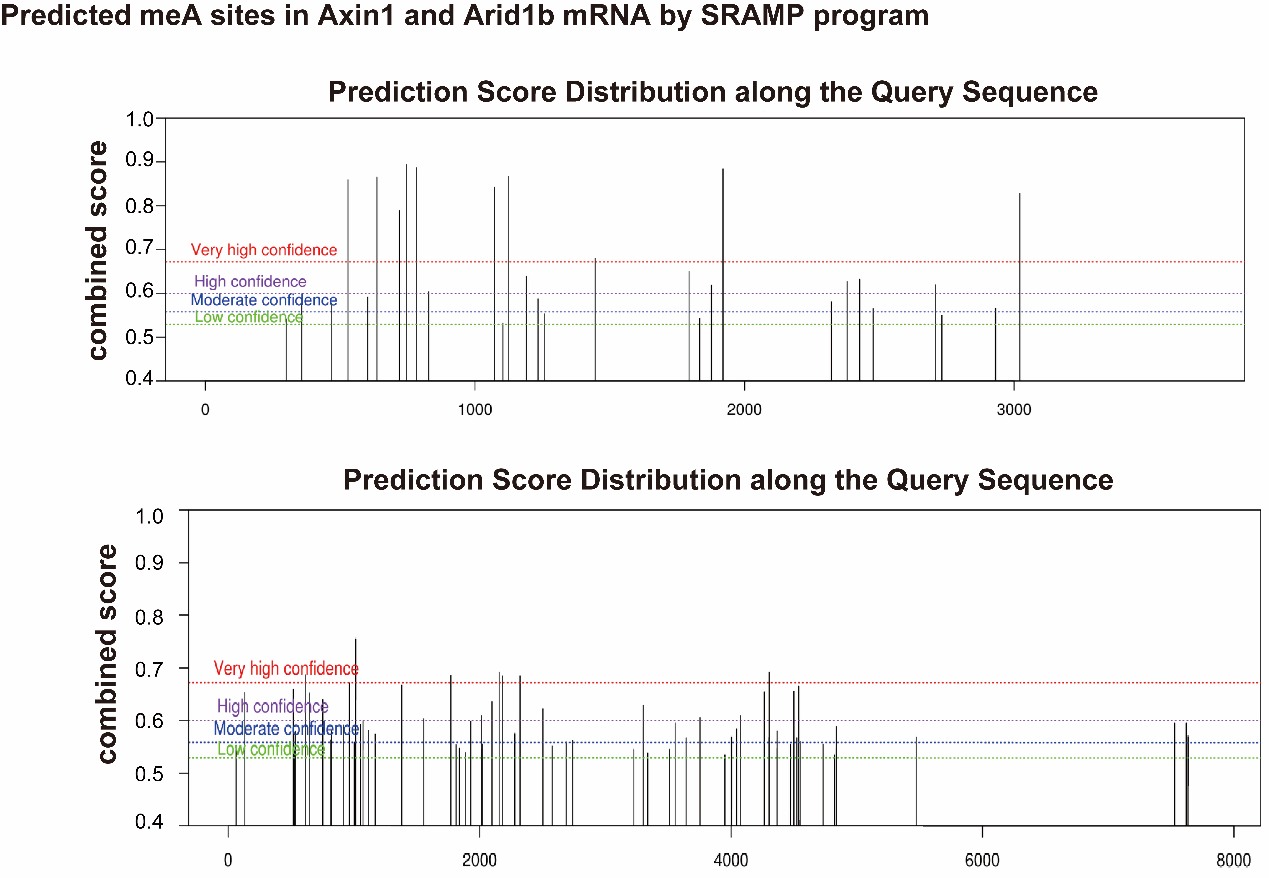
Figure S6:** **Prediction of m^6^A methylation sites of Axin1 and Arid1b using the SRAMP program**
